# Use of a Novel Detection Tool to Survey Orthohantaviruses in Wild-Caught Rodent Populations

**DOI:** 10.3390/v14040682

**Published:** 2022-03-25

**Authors:** Samuel M. Goodfellow, Robert A. Nofchissey, Chunyan Ye, Jonathan L. Dunnum, Joseph A. Cook, Steven B. Bradfute

**Affiliations:** 1 Center for Global Health, Department of Internal Medicine, University of New Mexico Health Sciences Center, Albuquerque, NM 87131, USA; sgoodfellow@salud.unm.edu (S.M.G.); rnofchissey@salud.unm.edu (R.A.N.); cye@salud.unm.edu (C.Y.); 2 Museum of Southwestern Biology, Biology Department, University of New Mexico, Albuquerque, NM 87131, USA; jldunnum@unm.edu (J.L.D.); cookjose@unm.edu (J.A.C.)

**Keywords:** detection, emerging, orthohantavirus, hantavirus, zoonosis, zoonotic pathogen, sequencing, PCR, cDNA synthesis

## Abstract

Orthohantaviruses are negative-stranded RNA viruses with trisegmented genomes that can cause severe disease in humans and are carried by several host reservoirs throughout the world. Old World orthohantaviruses are primarily located throughout Europe and Asia, causing hemorrhagic fever with renal syndrome, and New World orthohantaviruses are found in North, Central, and South America, causing hantavirus cardiopulmonary syndrome (HCPS). In the United States, Sin Nombre orthohantavirus (SNV) is the primary cause of HCPS with a fatality rate of ~36%. The primary SNV host reservoir is thought to be the North American deer mouse, *Peromyscus maniculatus*. However, it has been shown that other species of *Peromyscus* can carry different orthohantaviruses. Few studies have systemically surveyed which orthohantaviruses may exist in wild-caught rodents or monitored spillover events into additional rodent reservoirs. A method for the rapid detection of orthohantaviruses is needed to screen large collections of rodent samples. Here, we report a pan-orthohantavirus, two-step reverse-transcription quantitative real-time PCR (RT-qPCR) tool designed to detect both Old and New World pathogenic orthohantavirus sequences of the S segment of the genome and validated them using plasmids and authentic viruses. We then performed a screening of wild-caught rodents and identified orthohantaviruses in lung tissue, and we confirmed the findings by Sanger sequencing. Furthermore, we identified new rodent reservoirs that have not been previously reported as orthohantavirus carriers. This novel tool can be used for the efficient and rapid detection of various orthohantaviruses, while uncovering potential new orthohantaviruses and host reservoirs that may otherwise go undetected.

## 1. Introduction

Orthohantaviruses (order *Bunyavirales*, family *Hantaviridae*, subfamily *Mammantaivirnae*, genus *Orthohantavirus*) are enveloped, negative-sense RNA viruses that are carried by host reservoirs, such as rodents, shrews, and moles, throughout the world [1,2,3,4,5,6]. The two syndromes they cause in humans are hemorrhagic fever with renal syndrome (HFRS) and hantavirus cardiopulmonary syndrome (HCPS), caused by Old World and New World orthohantaviruses, respectively [2,7,8,9]. Old World orthohantaviruses are primarily found throughout parts of Asia, Africa, and Europe, while New World orthohantaviruses are located in North, Central, and South America. HFRS was first recognized in 1951 followed by the identification of the striped field mouse (*Apodemus agrarius*) as the source of infection in South Korea [7], subsequently encouraging the discovery of other host reservoirs with human transmission. There are now over 20 orthohantaviruses identified as causing illnesses in humans through transmission via wild rodents [10]. Seoul virus, whose host reservoirs are the Norway rat (*Rattus norvegicus*) and the black rat (*Rattus rattus*), has a worldwide distribution, since these rodents have been spread globally via ships for hundreds of years [11]. The first recognized outbreak of orthohantavirus infection in humans in the Western Hemisphere occurred in 1993 within the Four Corners region of the United States, in which Sin Nombre orthohantavirus (SNV) was first characterized [8,9]. SNV has a case mortality rate of ~36% and over 700 cases have been reported since its initial discovery, with New Mexico having had the most cases of HCPS [12]. Foundational work on SNV and the North American deer mouse’s (*Peromyscus maniculatus*) ecology in the US Southwest [13] has provided a framework for further study of orthohantavirus diversity, distribution, and evolutionary dynamics in wild host populations.

The primary host reservoir of SNV is thought to be the North American deer mouse (*Peromyscus maniculatus*) [14], but other *Peromyscus* species have been shown to carry different types of orthohantaviruses or variants of SNV that can cause HCPS in the US [15,16,17,18,19,20,21]. New York virus, now recognized as an SNV variant, caused outbreaks between 1993 and 1995, and is carried by the white-footed mouse (*P. leucopus*) [4,18,22]. While Limestone Canyon virus is carried by the brush mouse (*P. boylii*), there is also evidence from serum antibody testing that SNV can reside in the pinyon mouse (*P. truei*), the white-footed mouse (*P. leucopus*), and the cactus mouse (*P. eremicus*) [23,24]. Interestingly, all five of these *Peromyscus* species reside in close proximity in New Mexico; however, a detailed understanding of orthohantavirus distribution across this community remains elusive.

Tools such as enzyme-linked immunosorbent assay (ELISA) and nested PCR have been used to identify potential novel orthohantaviruses previously; nonetheless, rapid detection and screening are necessary to help expedite their discovery [25,26,27,28,29]. Reverse-transcription quantitative real-time polymerase chain reaction (RT-qPCR) has been shown to be a sensitive and specific method to rapidly identify orthohantaviruses in tissue [30,31,32,33]. In this study, we describe a rapid, novel pan-orthohantavirus two-step RT-qPCR tool that can be used in the detection of both known and potentially novel orthohantaviruses in wild-caught rodents.

## 2. Materials and Methods

### 2.1. Primer Design

Degenerate primers were designed based on multiple New and Old World orthohantaviruses using the N gene in the S segment of sequences available on the National Center for Biotechnology Information’s (NCBI) GenBank. These included: Sin Nombre (NC_005216), *Peromyscus maniculatus*; Andes (NC_003466.1), *Oligoryzomys longicaudatus*; Maporal (NC_034566.1), *Oligoryzomys fulvescens*; Dobrava-Belgrade (NC_005233.1), *Apodemus agrarius*; Seoul (AY273791), *Rattus norvegicus*; Hantaan (M14626), *Apodemus agrarius*; Puumala (X61035), *Myodes glareolus*; Tula (Z69991), *Microtus arvalis*; Muleshoe (KX066124), *Sigmodon* hispidus; Black Creek Canal (L39949.1), *Sigmodon hispidus*; Rio Mamore (KF584259), *Oligoryzomys microtis*; Bayou (NC_038298.1), *Oryzomys palustris*; Prospect Hill (M34011.1), *Microtus pennsylvanicus*; and Choclo (NC_038373), *Oligoryzomys fulvescens*. Sequences were compiled and aligned using the EMBL-EBI CLUSTAL Omega Multiple Sequence Alignment DNA tool [34]. All sets of primers (PanHS1, PanHS2, PanHS7, and PanHS8) were used to detect plasmid and authentic viruses. Screening of rodent samples was conducted using PanHS8. All primers were custom-designed and then synthesized by ThermoFisher. The primer sequences used in this study are listed in Table 1.

### 2.2. Plasmid Construction and Cultured Viruses

Plasmids for orthohantaviruses (Hantaan (HNTV), Seoul (SEOV), Black Creek Canal (BCCV), Bayou (BAYV), Rio Mamore (RIOV), Maporal (MAPV), Tula (TULV), Dobrava (DOBV), Sin Nombre (SNV), Muleshoe (MULV), Puumala (PUUV), Prospect Hill (PHV), and Andes Virus (ANDV)) were generated through GenScript using the N gene from the S segment reference sequence listed above for the virus and expressed in a pFastBac1 backbone. For authentic viruses, Vero E6 cells were infected with an MOI of 1 with either SNV/SN77734, ANDV/CHI-7013, HTNV/76-118, SEOV/Baltimore, PUUV/P360, PHV/PH-1, RIOV/LH 060/2011, BAYV/HV F0260003, or TULA/Moravia/5302v/95. Cells were washed with PBS in a BSL-3 facility followed by lysing, before RNA extraction with the QIAmp^®^ Viral RNA Mini Kit (Qiagen, Hilden, Germany) according to the manufacturer’s instructions at BSL-2 with BSL-3 practices.

### 2.3. Trapping and Sample Collection

Rodent samples, primarily *Peromyscus*, were collected from the following New Mexico counties: Valencia, Cibola, Torrance, Bernalillo, and Catron using Sherman live traps (38 × 3.59 × 239 cm H.B. Sherman Co., Tallahassee, FL, USA) baited with peanut butter and oats. Sites within Valencia and Bernalillo counties were in a mixture of residential and rural areas. All other sites were populated with either pinyon, ponderosa, and/or juniper trees. All field procedures were performed following the animal care and use guidelines of the American Society of Mammalogists [35] and approved by the University of New Mexico Institutional Animal Care and Use Committee, collected under the New Mexico Department of Game and Fish permit to J. A. Cook (authorization number 3300). Holistic museum specimens were prepared according to the best practices for emerging pathogen research and databased in a relational collection management system (Arctosdb.org) to facilitate the linkage of host specimen data and derived pathogen data, which include the exact latitude and longitude for each trap location [36,37]. Standard measurements (total length, tail length, hind foot (with claw), ear (from notch), weight, reproductive data (sex, reproductive status, testes, and embryo crown–rump measurements), and age were recorded. Species identifications were conducted through a combination of measurement data and morphological characters and confirmed through cytochrome *b* sequence analysis. The tissues collected were snap-frozen in N_2_ and included the brown fat, spleen, heart, lung, kidney, liver, colon (with feces), urinary bladder (w/urine if present), and serum from blood centrifuged in the field. Small mammal specimens were deposited at the Museum of Southwestern Biology, University of New Mexico (MSB), Division of Mammals (DOM), and Division of Genomic Resources (DGR). Specimens were cataloged with the following MSB:Mamm numbers: 329203–329212, 329247–329251, 329261, 329263–329271, 329277, 329281–329283, 329285–329288, 332704, 332712, 332713, 332717, 332718, 332720, 332721, 332723–332727, 332730, 332733, 332736–332740, 332743, 332744, 332758–332764, 332766, 332768–332783, 332785–332788, 332790–332804, and 332875–338877.

### 2.4. RNA Extraction

RNA extraction from rodent lung tissue was also performed using the QIAmp^®^ Viral RNA Mini Kit (Qiagen) according to the manufacturer’s instructions with slight modifications. A total of 40 mg of frozen lung tissue was homogenized using a BeadBug^TM^ 6 Microtube Homogenizer (Benchmark Scientific, Sayreville, NJ, USA) in a bead beater tube preloaded with 1.0 g of 1.0 mm-diameter zirconia beads (catalog number 1107911zx; BioSpec, Bartlesville, OK, USA), 1.0 g of 2.0 mm-diameter zirconia beads (catalog number 11079124zx; BioSpec), and 600 mL of AVL buffer. The tissue was beaten at 4350 rpm for 30 s for 1 cycle. Homogenates were then centrifuged, placed in a new 1.5 mL microcentrifuge tube, and re-centrifuged to remove any excess debris. The RNA carrier was then added to the cleaned lysate and cleanup proceeded via the manufacturer’s instructions.

### 2.5. Reverse Transcriptase-Quantitative Polymerase Chain Reaction (RT-qPCR) and Nested PCR

Reverse transcription (RT) using SuperScript II (Invitrogen, Waltham, MA, USA, Thermo Fisher Scientific, Waltham, MA, USA) was established for the QuantStudio5 series (Applied Biosystems, Waltham, MA, USA). First, the RT was performed using 5 μL of RNA (~500 ng) with 1 μL of SuperScript II containing 4 μL 5x First-Strand Buffer, 2 μL of 0.1 M DTT, 1 μL of RNaseOUT, 1 μL of random primers, 1 μL of dNTP Mix (10 mM), and 5 μL of RT-qPCR-grade water. This reaction was incubated at 65 °C for 5 min, placed on ice briefly, followed by 10 min at room temperature for binding with a 50 min reaction at 42 °C, then terminated by 15 min at 70 °C. The qPCR reactions were carried out by using 25 μL of POWER SYBR Green PCR Master Mix (Applied Biosystems, Thermo Fisher Scientific) containing 3 μL of cDNA (~300 ng), 2 μL of primer (5–10 μM), and 20 μL of DEPC-treated water (Ambion, Austin, TX, USA) in a 50 μL final volume reaction. For each sample, duplicate wells were tested using the following cycling conditions: 2 min at 50 °C followed by 10 min at 95 °C for the hold stage, while the PCR stage held for 15 s at 95 °C, the suggested annealing temperature according to Table 1 for 1 min for a total of 40 cycles, followed by a melt curve stage. The controls included a no-template control (NTC), in vitro transcribed RNA from SNV-infected Vero E6 cells, orthohantavirus plasmids, and either positive or negative rodent lung tissue samples. Additionally, β-actin primers (F-ATG TAC GTA GCC ATC CAG GC and R-TCT TGC TCG AAG TCT AGG GC) specific for *Peromyscus maniculatus* were used as an internal control [38]. For low viral expression, additional cycling (45 cycles) was suggested, along with diluted cDNA at 1:10 with nuclease-free water to remove potential inhibitors. An adjusted threshold was conserved across grouped experiments as well as an adjusted baseline cycle between 5 and 15 cycles.

Additionally, selected samples were also confirmed for an orthohantavirus using previously published pan-orthohantavirus primers (HAN-L-F1: 5′-ATGTAYGTBAGTGCWGATGC-3′ and HAN-L-R1: 5′-AACCADTCWGTYCCRTCATC-3′ for primary PCR, HAN-L-F2: 5′-TGCWGATGCHACIAARTGGTC-3′, and HAN-L-R2: 5′-GCRTCRTCWGARTGRTGDGCAA-3′) against the L segment through nested PCR using 300 ng of cDNA in 25 µL total volume reactions. First, PCR was conducted with HAN-L-F1/R1 using standard PCR conditions, but with an annealing temperature of 55 °C for 25–35 cycles. An amount of 3 µL of product from the first PCR was carried forward using HAN-L-F2/R2 with standard PCR conditions and with an annealing temperature of 59 °C, up to 40 cycles. All reactions were conducted using GoTaq Master Mix (Promega, Madison, WI, USA). The nested PCR products are shown in Supplementary Figure S1C.

### 2.6. DNA gel Electrophoresis

Additional PCR amplification was performed on positive samples screened using RT-qPCR as described above with 45 cycles. DNA gel electrophoresis was performed using 2% agarose gels prepared with SYBR^TM^ Safe DNA Gel Stain in 0.5X TBE (Invitrogen, Waltham, MA, USA), then run at 80 V (120 V dependent on the size of the gel) with a 100 bp DNA ladder and imaged using an Analytik Jena gel imager.

### 2.7. Sanger Sequencing

Positive pan-orthohantavirus samples were confirmed through Sanger Sequencing (GeneWiz, Plainfield, NJ, USA & Sequetech, Mountain View, CA, USA). PCR products were visualized through gel electrophoresis and cleaned up using a Qiagen QIAquick PCR Purification Kit (Cat. # 28106) or QIAquick Gel Extraction Kit (Cat. # 28704) according to the manufacturer’s instructions. Cleaned-up samples were then prepared according to the company’s submission instructions for pre-mix and the sequences obtained were then used in NCBI’s nucleotide Basic Local Alignment Search (BLAST) tool.

### 2.8. Software Programs

QuantStudio Design and Analysis Software v1.5.1 was used to analyze qPCR data. Figures and images were made using Prism (version 9.1.0, GraphPad, San Diego, CA, USA) and Illustrator (version CC 2019, Adobe, San Jose, CA, USA), and tables were generated using Word (version 2016, Microsoft, Redmond, WA, USA). The graphical summary was created by BioRender.com (Agreement number *AD23PQ41AM)*.

## 3. Results

### 3.1. Primer Design for Pan-Orthohantaviruses Detection

The orthohantavirus nucleocapsid (N) gene encoded in the S segment was shown to be expressed at high levels during early serological response, and although it was not as conserved as the RNA-dependent RNA polymerase (RdRp), it was an ideal target for broad detection of orthohantavirus species [39,40]. Primers were designed by compiling and aligning 14 New and Old World pathogenic orthohantavirus S segment sequences available in the National Center for Biotechnology Information (NCBI) GenBank. About 76% identity was observed, and variable nucleotide sites were completed with degenerate nucleotides, resulting in eight sets of primer pairs.

### 3.2. Validation of Pan-Orthohantavirus Primers for Detection of Both New and Old World Orthohantaviruses

**Figure 1:**
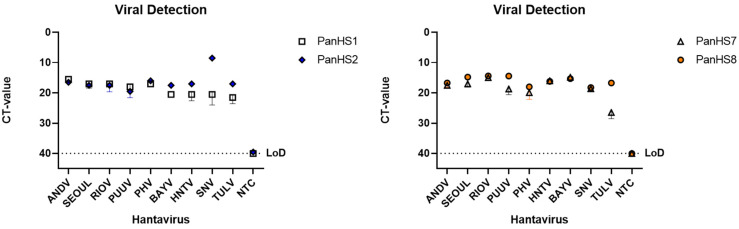
Plasmids from both Old and New World orthohantaviruses are detected by pan-orthohantavirus primers. Amplification CT values for the SYBR green two-step RT-qPCR assay are depicted. Primers were tested against designed plasmids with the sequence of the N gene from the S segment of both New and Old World orthohantaviruses in a pFastBac1 vector. Thresholds were automatically assigned along with the baseline cycle. Hantaan (HNTV); Seoul (SEOV); Black Creek Canal (BCCV); Bayou (BAYV); Rio Mamore (RIOV); Maporal (MAPV); Tula (TULV); Dobrava (DOBV); Sin Nombre (SNV); Muleshoe (MULV); Puumala (PUUV); Prospect Hill (PHV); Andes Virus (ANDV). CT: Cycle threshold. Limit of detection (LoD). Representative of three independent experiments.

**Figure 2:**
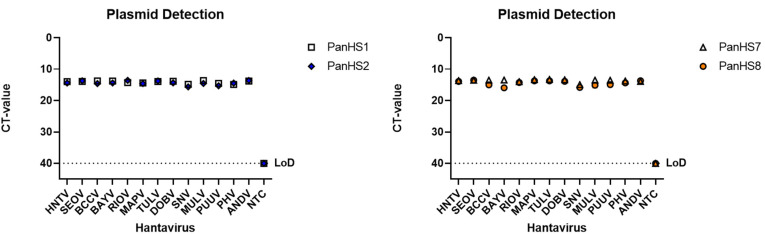
Detection of New and Old World authentic orthohantaviruses by pan-orthohantavirus primers. Amplification CT values for the SYBR green two-step RT-qPCR assay are depicted. Primers were tested against both New and Old World orthohantaviruses cultured in vitro. Thresholds were automatically assigned along with the baseline cycle. Andes Virus (ANDV); Seoul (SEOV); Rio Mamore (RIOV); Puumala (PUUV); Prospect Hill (PHV); Bayou (BAYV); Hantaan (HNTV); Sin Nombre (SNV); Tula (TULV). CT: Cycle threshold. Limit of detection (LoD), Representative of three independent experiments.

To validate these pan-orthohantavirus primer pairs, we used an SYBR green qPCR assay to test them against plasmids carrying the N gene from Old and New World orthohantaviruses. We found that, of the eight initial primer sets designed, four (PanHS1, PanHS2, PanHS7, and PanHS8; Table 1) were the most consistent and sensitive in the detection of N in plasmids across 13 orthohantaviruses: Hantaan (HNTV), Seoul (SEOV), Black Creek Canal (BCCV), Bayou (BAYV), Rio Mamore (RIOV), Maporal (MAPV), Tula (TULV), Dobrava (DOBV), Sin Nombre (SNV), Muleshoe (MULV), Puumala (PUUV), Prospect Hill (PHV), and Andes (ANDV) (Figure 1). Next, we tested whether the primer sets could detect authentic New and Old World orthohantaviruses. We tested RNA extracted from nine orthohantaviruses (ANDV, SEOV, RIOV, PUUV, PHV, BAYV, HNTV, SNV, and TULV) and confirmed detection with each set of primers (Figure 2). We found that single bands were visible on an agarose gel for each of the four sets of primers when testing against plasmids and viruses, suggesting a single product (Supplementary Figure S1A,B). We found PanHS8 to be the most consistent between replicate samples and used this primer set as our primary tool for screening orthohantavirus in wild-caught rodent samples.

### 3.3. Screening Wild-Caught Rodents Using Pan-Orthohantavirus Primers

**Figure 3:**
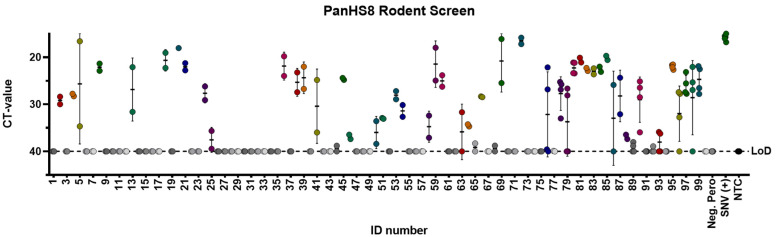
PanHS8 detects orthohantaviruses other than Sin Nombre virus (SNV) in wild-caught animals. Amplification CT values for the SYBR green two-step RT-qPCR assay are depicted. PanHS8 was selected and tested against cDNA from the lung tissue of 100 wild-caught animals. Positive PanHS8 samples are shown in color. Thresholds were adjusted to where the amplification efficiency peaked, and the baseline cycle was adjusted to 5 to 15 cycles. SNV virus and confirmed negative wild-caught rodents were used as controls. Beta actin was tested in parallel as a control. Sample IDs were replaced with a rodent ID for plotting purposes. Each set of dots is represented by a standard error bar and mean. CT: Cycle threshold. Limit of detection (LoD).

**Figure 4:**
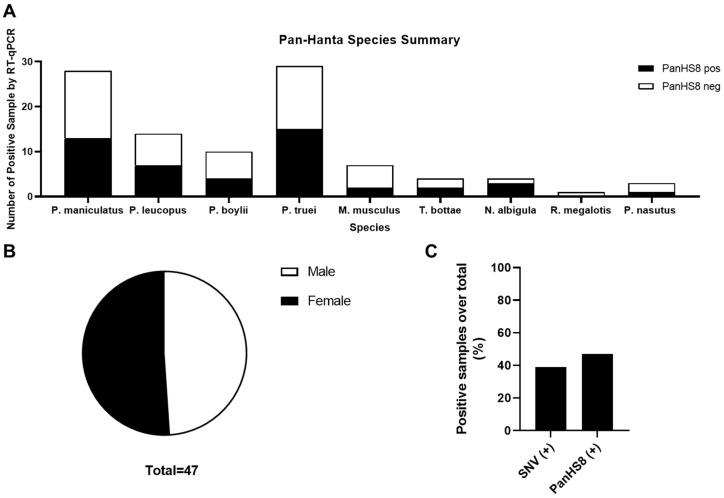
Summary of rodent species positive for PanHS8. Overall results indicating several species of *Peromyscus* along with additional rodents carrying orthohantavirus. (**A**) Positive rodents by species and genus for PanHS8 (black) compared to negative PanHS8 samples (white). (**B**) Sex was calculated using the total rodents positive for PanHS8. (**C**) Summary of the total percentage of positive samples by both SNV TaqMan and PanHS8 for an orthohantavirus.

With a validated tool set capable of detecting both Old and New World orthohantaviruses in plasmids and authentic viruses, we further tested this approach in a screen of lung tissue from wild-caught rodents (primarily five *Peromyscus* species, but also four other rodent species) in New Mexico. We performed a screen of homogenized lung tissue from 100 wild-caught rodents and detected orthohantavirus in 47 rodents (47% of the samples tested) (Figure 3 and Figure 4A). We detected orthohantavirus in each *Peromyscus* species tested (*P*. *maniculatus*, *P. leucopus*, *P. boylii*, *P. nasutus* and *P. truei*) (Table 2). We also detected transcripts for orthohantavirus in the common house mouse (*Mus musculus*) and, for the first time, in both Botta’s pocket gopher (*Thomomys bottae*) and the white-throated woodrat (*Neotoma albigula*) (Figure 4A and Table 2). Overall, we found that there was an almost equal ratio of males to females of total rodents carrying an orthohantavirus detected with PanHS8 (Figure 4B). Rodents were also specifically tested for SNV by using a published two-step Taqman RT-qPCR method [41]. We found that 39% of the trapped rodents carried SNV (Figure 4C). Interestingly, we found a subset of rodents that were positive by PanHS8 but negative for SNV (Figure 4C). The high rate of orthohantavirus positive rodents was an unanticipated result. Therefore, to ensure the accuracy of these primer sets for the screening of wild-caught rodents, we blindly repeated the testing of a subset of these samples (73 from the original 100) using different laboratory staff. This included fresh preparation of cDNA, RT-qPCR, and analysis, which was then compared to the original screening. We found that we were able to replicate ~88% of the original results, with an accuracy of 82.6% for positive samples and 90% accuracy for negative samples (Table 3); these findings reinforce the efficacy and reproducibility of this tool for screening orthohantaviruses in wild-caught rodents.

### 3.4. Sequencing PCR Fragments of Positive Rodents to Confirm Orthohantavirus Infection

To further examine the accuracy of orthohantavirus detection, we conducted Sanger sequencing using PanHS8 primers. In addition, we took a subset of the positive samples and performed nested PCR using a previously published primer set against a conserved L segment region [28] that has been widely used to detect orthohantaviruses. This is because we wanted to further validate our positive samples with an established method and sequence a portion of the L segment that is known to be more conserved, but may not always be as highly expressed. We confirmed that, in wild-caught rodents, a band of the predicted amplicon size was present for 96% of the positive samples screened with PanHS8 (Supplementary Figure S1C). However, samples positive for PanHS8 did not always amplify using the L primer sets, but only a subset did (Supplementary Figure S1D) [28]. PCR products were then subjected to Sanger sequencing. Of all positive PanHS8 samples we sequenced 47, and found that 45 samples had an orthohantavirus sequence as the first match using NCBI nBLAST, with only two samples resulting in a false positive, demonstrating a specificity of 96% for PanHS8 (Table 4).

## 4. Discussion

**Figure 5:**
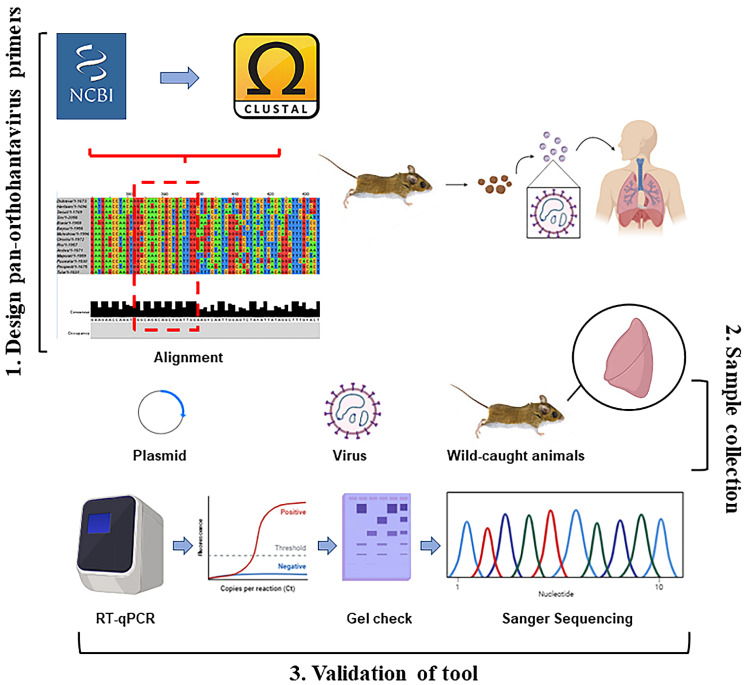
Overall summary of two-step RT-qPCR pan-orthohantavirus detection tool. 1. S segment sequences from both New and Old World orthohantaviruses were compiled and aligned through NCBI and CLUSTAL Omega. Little to no completely conserved regions existed; therefore, degenerate primers were designed. 2. Samples (plasmids, in vitro cultured virus, and lung tissue from wild-caught rodents) were used to validate primer selection. 3. Two-step RT-qPCR was performed followed by DNA gel electrophoresis. Sanger sequencing was also used for additional confirmation in positive lung tissue samples from wild-caught rodents.

Dozens of orthohantaviruses have been reported since the initial discovery of Hantaan orthohantavirus in 1978, with ongoing studies clarifying their host reservoirs and impact on human health [1,2,3,7,19,42]. The dearth of vaccines or therapeutics for either the prevention or treatment of orthohantavirus disease indicates a critical need to identify, characterize, and monitor these viruses across diverse wild reservoirs. Here, we report a rapid, effective two-step RT-qPCR detection tool that can be used across various platforms to detect known and potentially novel orthohantaviruses in a quantitative fashion (Figure 5). Although the wild rodent sampling in this study was performed in New Mexico, our plasmid and virus data suggest that this primer set could also be utilized elsewhere, in]cluding for testing Old World orthohantaviruses (Figure 1 and Figure 2).

Existing detection platforms have been widely used to identify known and novel orthohantaviruses while playing a vital role in our understanding of these viruses. For example, ELISAs have been employed to detect antibody responses against orthohantaviruses [25,26,43,44]. However, that approach does not typically distinguish between active or past infection and does not allow for the identification of the orthohantavirus due to antigen cross-reactivity [43]. Methods to perform indirect immunofluorescence assays (IFA) for multiple orthohantaviruses have also been developed, but those methods have been used mostly for diagnosing orthohantavirus infections, rather than for screening tissues [45].

In addition to antibody-based detection, nested and semi-nested RT-PCR assays are widely used and well-established methods for orthohantavirus detection [28]. These primers are typically designed against the more conserved polymerase gene (L segment); however, the N gene is expressed at higher levels [39]. Furthermore, nested RT-PCRs require two reactions, while RT-qPCR can give a rapid result and is more amenable to screening a larger number of samples [30,46,47,48]. We also acknowledge that there are existing RT-qPCR assays for detection, but they are specifically for Old World orthohantaviruses or individual orthohantaviruses, which may under-report the numbers of potential host reservoirs and infections [31,32,49,50,51,52,53,54]. We overcame the challenging task of designing primers for multiple pathogenic orthohantaviruses against the more highly expressed, but less conserved, nucleocapsid gene (S segment) by using highly degenerate primers. Interestingly, we found many rodents that were SNV-positive but negative for PanHS8. This could occur due to the lower sensitivity of SYBR green used with the degenerate PanHS8 primers compared to the high sensitivity of the specific SNV TaqMan probe. It is possible that mutations in the primer binding regions could also affect detection. Therefore, the use of multiple primer sets should be considered when screening rodents for orthohantaviruses.

To assess whether our primers could have non-specific binding, we screened the primers using a thermonucleotide BLAST program and found very rare cross-reactive sequences. Two were regions for the chromosomes of Bighorn sheep (*Ovis canadensis canadensis*) isolates and the Gram-negative bacterium *Magnetospirillum gyrphiswaldense*. These sequences producing false positives does not seem likely and provides additional confidence to the specificity of the target region of these primers.

This tool can be used to not only detect known orthohantaviruses, but also to potentially discover novel orthohantaviruses. These sequences identified in Table 4 are for both SNV and non-SNV hantaviruses, but further experiments are needed to conclusively identify the viruses found in this study. Although we cannot define a novel orthohantavirus by using a single short PCR fragment, our samples can be followed up by using metatranscriptomics to generate complete genomes [55,56,57]. Recently, orthohantaviruses, such as Bruges virus in the European mole (*Talpa europaea*) and Đakrông virus in Stoliczka’s Asian trident bats (*Aselliscus stoliczkanus*), have been found through nested PCR using pan-orthohantavirus L primers [29,31]. Aside from orthohantaviruses, we demonstrate that potential novel hosts, such as *T. bottae* and *N. albigula*, can be discovered using the novel PanHS8 primer set. Further studies need to be conducted to determine whether those species carry an infectious virus and if they can transmit to other animals. Additionally, incorporating wider mammalian diversity, including shrews, bats, and other hosts, would further test and potentially expand the utility of this approach.

Together, these results suggest that this is an effective two-step RT-qPCR pan-orthohantavirus detection tool for use in screening large numbers of wild-caught rodent specimens. This approach will contribute to furthering the understanding of orthohantaviruses and host reservoirs, work that has expanded greatly since the discovery of novel orthohantaviruses in rodents, shrews, and moles, and can allow for the enhancement of the surveillance of potential emerging zoonotic diseases.

## Figures and Tables

**Table 1:** Primers designed for the two-step RT-qPCR for pan-hantaviruses against the N gene of the S segment.

Primer		Sequence (5′-3′)	Target Segment (Gene)	Size (bp)	T^M^
PanHS1	Forward	GGRCARACHGCWGAYTGG	S (N)	248	62 °C
	Reverse	CCDGGHGTBADYTCHTCDGCYTTCAT			
PanHS2	Forward	GAYATGMGDAAYACNATHATGGC	S (N)	207	61 °C
	Reverse	CWGGRTCCATRTCATCHCC			
PanHS7	Forward	GGVCARACMGCWGAYTGG	S (N)	248	57 °C
	Reverse	CCWGGTGTNADYTCWTCDGC			
PanHS8	Forward	CAGGAYATGVGRAAYACVATHATGGC	S (N)	210	63 °C
	Reverse	CTCWGGRTCCATRTCATCMCC			

Mixed bases: B = G, T, C; D = G, A, T; H = A, T, C; K = G, T; M = A, C; N = A, T, G, C; R = A, G; W = A, T; V = A, C, G; and Y = C, T.

**Table 2:** Summary of 100 wild-caught rodents throughout New Mexico and tested for PanHS8. Sex M/F represents the percentage of orthohantavirus-positives for each male (M) and female (F) rodent.

Genus Species	# Screened (%)	Sex M/F (% +)	Lung Tissue
			PanHS8 + (%)
*Peromyscus maniculatus*	28 (28%)	16 (50%)/12 (42%)	13 (46%)
*Peromyscus leucopus*	14 (14%)	8 (38%)/6 (67%)	7 (50%)
*Peromyscus truei*	29 (29%)	13 (38%)/16 (63%)	15 (52%)
*Peromyscus boylii*	10 (10%)	6 (33%)/4 (50%)	4 (40%)
*Peromyscus nasutus*	3 (3%)	1 (0%)/2 (50%)	1 (33%)
*Mus musculus*	7 (7%)	5 (40%)/2 (0%)	2 (29%)
*Thomomys bottae*	4 (4%)	2 (100%)/2 (0%)	2 (50%)
*Neotomas albigula*	4 (4%)	2 (100%)/2 (50%)	3 (75%)
*Reithrodontomys megalotis*	1 (1%)	0 (0%)/1 (0%)	0 (0%)
Total(s)	100 (100%)	53 (45%)/47 (49%)	47 (47%)

**Table 3:** Summary of the independent and replicated results for the pan-orthohantavirus screening tool. Samples were prepared, run, and analyzed independently for the validation of the screening tool and then compared to the original screening results.

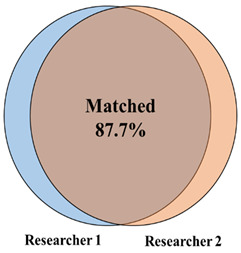		
**Repeated RT-qPCR Samples**	**Positive Percentage Accuracy**	**Negative Percentage Accuracy**
**Total (n = 73)**	82.6%	90.0%

**Table 4:** Results from pan-orthohantavirus screening using RT-qPCR. Positives were confirmed using Sanger sequencing and NCBI BLAST for the orthohantavirus result. A total of 100 samples were tested.

Rodent Samples Results	Number (%) of Hantavirus-Negative or Positive Rodents
Negatives	53 (53%)
False positives	2 (2%)
Positives	45 (45%)
Total	100 (100%)

## Supplementary Materials

The following supporting information can be downloaded at: https://www.mdpi.com/article/10.3390/v14040682/s1, Figure S1. DNA gel electrophoresis of RT-qPCR and nested PCR products. The 2% gels were run at either 80 V or 120 V and then imaged to visualize products from PCR runs. (A) All pan-orthohantavirus primers were tested against plasmids. SNV and ANDV are shown along with an NTC from one of the primer sets. Bands are the expected size according to Table 1. (B) The indicated primers were examined using in vitro cultured SNV, ANDV, and PHV. (C) PanHS8 primers amplified ~200 bp single-band products from rodent lung tissue. (D) HanL2 primers against the L segment were used in a handful of samples for nested-PCR (28). Bands were amplified according to ~400 bps as suggested and were then sequenced. All gels were run with a 100 bp DNA ladder.
