# Supplementary material for: Use of a Novel Detection Tool to Survey Orthohantaviruses in Wild-Caught Rodent Populations
Source: Viruses. 2022 Mar 25;14(4):682. doi: 10.3390/v14040682 (PMC9024935; doi:10.3390/v14040682)
Supplement: Supplementary file 1 [file viruses-14-00682-s001.zip › 1.pdf]

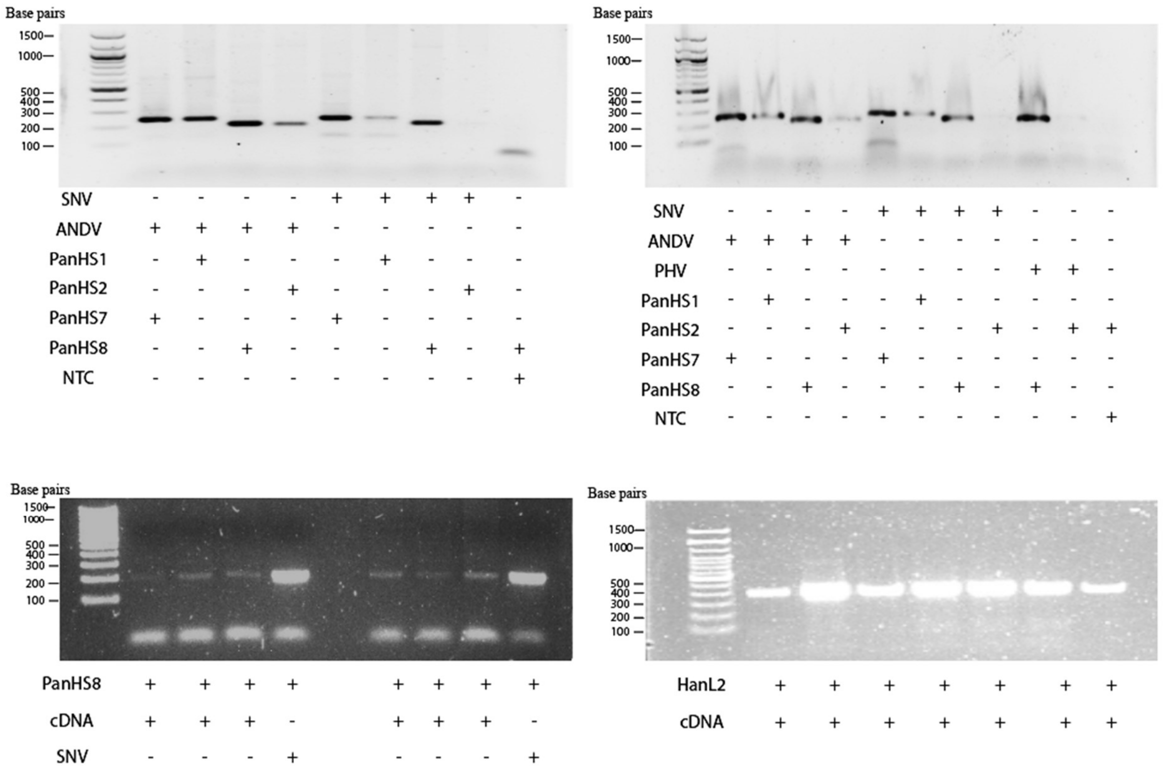

Supplemental Figure S1. DNA gel electrophoresis of RT-qPCR and nested PCR products. 2% gels were run at either 80V or 120V then imaged to visualized products from PCR runs. A) All pan-orthohantavirus primers were tested against plasmids. SNV and ANDV are shown along with an NTC from one of the primer sets. Bands are expected size according to Table 1. B) The indicated primers were examined using *in vitro* cultured SNV, ANDV and PHV. C) PanHS8 primers amplified ~200 bp single band products from rodent lung tissue. D) HanL1 and HanL2 primers against the L segment were used in a handful of samples for nested-PCR (28). Bands amplified according to ~400 bps as suggested. All gels were run with a 100 bp DNA ladder.
